# Second-line chemotherapy rechallenge in lung cancer patients: a Moroccan real-world study

**DOI:** 10.3389/fonc.2025.1489327

**Published:** 2025-05-22

**Authors:** Hassan Abdelilah Tafenzi, Farah Choulli, Ismail Essadi, Rhizlane Belbaraka

**Affiliations:** ^1^ Medical Oncology Department, Mohammed VI University Hospital of Marrakech, Marrakech, Morocco; ^2^ Biosciences and Health Laboratory, Faculty of Medicine and Pharmacy, Cadi Ayyad University, Marrakech, Morocco; ^3^ Medical Oncology Department, Avicenna Military Hospital of Marrakech, Marrakech, Morocco

**Keywords:** lung cancer, real world data (RWD), chemotherapy rechallenge, Moroccan cohort, second-line

## Abstract

**Background:**

Immune checkpoint inhibitors are the go-to therapeutic option for relapsed non-small cell lung cancer (NSCLC) with unidentified oncogenic drivers when first-line platin doublet chemotherapy fails. Meanwhile, few options exist for the treatment of relapsed patients with small cell lung cancer (SCLC). Through the present study, we evaluate the efficacy and hematologic safety of rechallenging chemotherapy in the second line after the failure of platinum-based chemotherapy.

**Methods:**

In this retrospective study, we selected patients admitted to a single institution. Adults aged > 18 years with a pathologically proven diagnosis of either NSCLC or SCLC, a PS of 2 or lower, and whose disease progressed during or after a platin-based doublet chemotherapy-containing line of treatment were eligible. The primary outcomes were second-progression-free survival (PFS) and overall survival (OS). Secondary endpoints included the proportion of patients with an overall response (complete or partial response), the disease control rate (DCR), and hematological safety.

**Results:**

Between January 2013 and December 2022, 155 patients were enrolled and treated in the second line with different available regimens of whom 145 had NSCLC and 10 had SCLC. As of December 31^st^, 2022, the median follow-up for the entire cohort was 4.6 [IQR: 2, 9.1] months. Overall, in the NSCLC patients, there was no statistical significance between the tested second-line regimens; the median PFS was 4.5 (95% CI: 3.6, 6.2) months (hazard ratio for progression: 1.1; 95% CI: 0.65, 1.86; p = 0.78), and the median OS was 10 (95% CI: 7.8, 16) months (hazard ratio for death: 1.49; 95% CI: 0.63, 3.54; p = 0.4). For the SCLC patients, we noticed the absence of statistical significance between treatment groups; the median PFS was 5.1 (95% CI: 1.9, Not Estimable [NE]) months (hazard ratio for progression: NE; p = 0.06), while statistical significance has been noticed between treatment groups in terms of proving OS; 5.1 (95% CI: 1.9, NE) months (hazard ratio for death: NE; p = 0.03). The overall response rate has not been reached (complete response = 0%; 2 patients have a partial response), and the disease control rate was 6.9% (n = 9) in the NSCLC population and 20% in the SCLC population. The most common grade 3–4 adverse hematological abnormalities were anemia (n = 30, 19.2%), neutropenia (n = 19, 12.3%), and thrombocytopenia (n = 14, 9.1%).

**Conclusion:**

At progression during or after first-line chemotherapy plus platinum, re-challenging single-agent chemotherapy in monotherapy or erlotinib did not offer modest activity in the Moroccan population.

## Introduction

In the past five years, there have been significant advancements in the first-line treatment of lung cancer (LC): the adoption of platinum-doublet combinations and immunotherapy as the gold standard for treating non-small cell LC (NSCLC) that has not received prior treatment has improved response rates, progression-free survival (PFS), and overall survival (OS) (1–5). The same improvement has been witnessed with small cell LC (SCLC) patients when adding monoclonal antibodies against programmed death 1 (PD-1) and its ligand PD-L1 to chemotherapy plus platinum in front-line with IMpower133 (6), CASPIAN (7), and KEYNOTE-604 (8).

However, nearly all patients, regardless of their stages, will experience either disease progression or clinical deterioration, which will require either further systemic therapeutic lines or won’t receive any treatment, and therefore will be oriented towards the best supportive care (BSC). Pivotal studies highlight that a small proportion of patients, generally less than 50%, are able to receive subsequent therapies after tumor progression under platin-based chemotherapy. For patients with NSCLC with unknowing molecular aberrations, after the failure of first-line chemotherapy, patients will still be offered immune checkpoint inhibitors (ICI) in the second-line treatment, while for SCLC tumors, the standard regime still includes chemotherapeutic agents. Nevertheless, in developing countries that still cannot afford targeted therapies for their patients, re-challenging different chemotherapy agents, whether they contain platinum or not, is the only hope.

In patients who have previously received platinum-based chemotherapy, docetaxel has been found to alleviate disease-related symptoms, extend progression, and improve overall survival (OS) compared to optimal supportive care (9).

Hence, through the following study, we aim to compare second-line treatment options for improving second PFS and OS, which compare the efficacy and hematologic safety of docetaxel with that of erlotinib and other chemotherapy agents in patients regardless of molecular aberrations that progressed under the combination of chemotherapy and platinum. Here, we report the results of the analysis.

## Methods

### Data source

Clinical data from one academic medical center that geographically covers middle and southern Morocco and serves more than 25% of the Moroccan population was used for this study (10). Patients diagnosed with all different stages of LC were identified from the Medical Oncology Department registry, from which we obtained all patients’ relevant data regarding their characteristics, vital status, and diagnosis.

In addition to patient-related data, pharmacotherapy information such as medication name, administration date, dosage, and route of administration was manually retrieved from each patient’s medical record to provide an overview of all employed regimens in the research population.

### Study population

Patients with different disease stages (IB-IVB) diagnosed between January 1, 2013, and December 31, 2022, were selected for the study. To be included, patients have to be aged beyond 18 years, pathologically or cytologically confirmed lung carcinoma in which the histologic tests revealed transitional-cell characteristics, have proven disease progression following platinum-based chemotherapy, or have relapsed within two years after platinum-based adjuvant or neoadjuvant therapy for advanced and localized disease, respectively, as assessed by Response Evaluation Criteria in Solid Tumors (RECIST), version 1.1 (11), and an Eastern Cooperative Oncology Group (ECOG) performance status (PS) score of 0, 1 or 2 on a scale of 5-point (12), to define the fits and ability of patients to receive further cycles at the beginning day of treatment. Patients with ECOG PS superior to 2 with a disability to carry out normal daily activity or incapable of self-care and had more than two extra-thoracic lesions or unknown first-line received regimens were excluded from enrolment.

The recorded patient characteristics include age at diagnosis; gender; tobacco status quantified by P/A; alcohol status; cannabis status; histology type (non-small cell vs. small-cell); clinical T, N, M classification; stage at diagnosis; initial brain metastasis; initial bone metastasis, initial liver metastasis, initial adrenal metastasis; ECOG PS; programmed death-ligand 1 (PDL-1); anaplastic lymphoma kinase (ALK); epidermal growth factor receptor (EGFR); also the therapeutic strategy of the first line treatment; second line chemotherapy regimens received; prior radiotherapy; and information on hematologic related adverse events from anemia, neutropenia, and thrombocytopenia were collected.

The staging, as well as the T, N, and M classifications, followed the last edition, the eighth edition of the American Joint Committee on Cancer (AJCC) system (13, 14).

### Identification of systemic treatments per patient

Second-line treatment is defined as systemic therapy used following first-line therapy-based platinum doublet chemotherapy completion or cessation due to disease progression. The investigator’s choice of the second-line treatment depended mainly on the drug availability in the department, previous first-line treatments administered, and the patient’s general condition.

The assignment has been divided based on administered regimens in which patients have received either erlotinib at a dose of 150 mg once daily, irinotecan at a dose of 350 mg per square meter of body-surface area on day one, docetaxel at a dose of 75 mg per square meter of body-surface area on day one, vinorelbine at a dose of 30 mg per square meter of body-surface area on days 1 and 8, gemcitabine at a dose of 1000 mg per square meter of body-surface area on days 1 and 8, paclitaxel at a dose of 175 mg per square meter of body-surface area on day one, pemetrexed at a dose of 500 mg per square meter of body-surface area on day one, and etoposide at a dose of 100 mg per square meter of body-surface area on days 1 and 8. Patients who benefited from an adjusted dose at the second line due to severe, untolerated toxicities were excluded from the study (**Supplementary Table S1** in the **Supplementary Appendix**).

Patients with disease progression as determined by computed tomography (CT) scan results and a clinically stable status received further line therapies, while patients who deteriorated during the second line after one cycle were oriented to BSC. Furthermore, patients who had a stable disease beyond six cycles without clinical disability who stopped or discontinued treatment were included.

### End points

PFS and OS were the primary evaluated endpoints in the total population. The period of time from the beginning of second-line treatment to death from any cause was referred to as the OS. The period of time from the beginning of the second treatment to the radiological progression of the disease, clinical progression, or death from any cause was referred to as PFS.

Efficacy, defined as the objective response rate, which included the percentage of patients with a confirmed complete or partial response to disease, was the second endpoint assessed for the entire population. All of the patients who had received at least one dosage of treatment were included in the group that was evaluated for hematologic safety as-treated, which was another secondary endpoint. Hematologic toxicities were the only relevant adverse events that were manually collected from the patient’s medical records to define the safety profile, as most patient medical records do not provide enough information regarding adverse events related to therapies. The National Cancer Institute Common Terminology Criteria for Adverse Events, version 5.0 stetted criteria, were followed to report the described hematologic-related adverse events (15).

### Statistical analysis

The Kaplan-Meier method (16) was used to estimate the time-to-event distribution of OS and PFS. In cases of lost follow-up, patients were traced using the registered phone number in their medical records. Nevertheless, some patients are not joinable, so in the OS analysis, these patients were left censored at the time of the last contact, while in the PFS analysis, patients who were alive and experiencing no disease progression or who were lost to follow-up had their data censored at the time of the most recent tumor assessment for the analysis of progression-free survival. The log-rank test (17) was adopted to calculate the between-group differences in OS and PFS. The analysis of the extra secondary or other outcomes was not part of the study’s hypothesis testing. A stratified Cox proportional-risks model (18) and Efron’s approach (19) to handling ties were used to determine the hazard ratios and related 95% confidence intervals. In order to compare treatment groups across variables, the Kruskal-Wallis rank sum test (20) and Fisher’s exact test (21) were employed. The receiver operating characteristic (ROC) (22) was adopted to determine the optimal cut-off value of a continuous variable that is strongly related to the event of interest. The cutoff for the database was December 31, 2022. All statistical analyses were performed using R software (version 4.1.2; R Foundation for Statistical Computing, Vienna, Austria).

## Results

### Baseline patients characteristics and treatment

We identified a total of 155 patients. Between January 2013 and December 2022, of all 1200 LC-diagnosed patients, 207 progressed after first-line platinum-containing chemotherapy, and only 155 were fitted to receive second-line therapy, divided into 145 patients with NSCLC histology and 10 patients with SCLC histology (**Supplementary Figure S1**, **Supplementary Appendix**).

In the NSCLC group of patients, 50 of 145 patients received docetaxel, 31 patients received gemcitabine, 26 patients received vinorelbine, 16 patients received paclitaxel, 17 patients received pemetrexed, and 5 patients received erlotinib. We should note that NSCLC patients who received erlotinib in the second-line therapeutic window were not selected according to EGFR status. Led by male patients (124 of 145), being heavy smokers 84 (63%), and no particular comorbidity sign (Charlson score = 0), diagnosed initially at stage IVA 52 (36%) with the presence of pleural effusion at 35 (24%) of all cases. The median age at diagnosis for the group was 58 [IQR: 52, 63] years. Following neo/adjuvant or palliative first-line platinum doublet chemotherapy, the common progression site was the lung; in addition, 46 (31.7%) of the selected patients received prior concomitant chemoradiotherapy. Approximately one-third of the patients, 39 (27%), had investigator-reported bone metastases at the time of diagnosis; 29 (20%) reported adrenal metastasis; 18 (12%) had liver metastasis; and 19 (13%) presented with brain metastasis. The same percentage of patients have previously undergone local brain irradiation. At the database lock (December 31, 2022), no patient from this group was continuing treatment. The median duration of prior second-line treatment and progression was 0.87 [IQR: 0.4, 1.6] months. The median duration between the last cycle of the first-line treatment and the beginning of second-line treatments was 1.4 [IQR: 0.5, 6] months. The median follow-up for the NSCLC group was 4.7 [IQR: 2.1, 9.8] months (**Table 1**).

**Table 1:** NSCLC patient characteristics.

Variables	Overall, N = 145*^1^*	Treatment Received						p-value*^2^*
		Docetaxel, N = 50*^1^*	Gemcitabine, N = 31*^1^*	Vinorelbine, N = 26*^1^*	Paclitaxel, N = 16*^1^*	Pemetrexed, N = 17*^1^*	Erlotinib, N = 5*^1^*	
Sex - no.(%)								0.12
Female	21 (14%)	5 (10%)	3 (9.7%)	4 (15%)	3 (19%)	3 (18%)	3 (60%)	
Male	124 (86%)	45 (90%)	28 (90%)	22 (85%)	13 (81%)	14 (82%)	2 (40%)	
Prior First-line regimens based platinum - no.(%)								0.87
Docetaxel	1 (0.7%)	0 (0%)	1 (3.2%)	0 (0%)	0 (0%)	0 (0%)	0 (0%)	
Gemcitabine	20 (13.4%)	9 (18%)	1 (3.2%)	4 (15.4%)	5 (31%)	0 (0%)	0 (0%)	
Navelbine	48 (33%)	22 (44%)	12 (39%)	2 (7.7%)	5 (31%)	6 (35%)	1 (20%)	
Paclitaxel	62 (43%)	14 (28%)	15 (48%)	20 (77%)	2 (12%)	10 (59%)	1 (20%)	
Pemetrexed	13 (9.0%)	5 (10%)	2 (6.5%)	0 (0%)	3 (19%)	1 (5.9%)	2 (40%)	
Erlotinib	1 (0.7%)	0 (0%)	0 (0%)	0 (0%)	1 (6.2%)	0 (0%)	0 (0%)	
Age at diagnosis - yr Median (IQR)	58 (52, 63)	58 (53, 63)	59 (56, 65)	60 (53, 64)	54 (53, 58)	54 (46, 58)	52 (45, 58)	0.030
Tobocco status - no.(%)								0.055
Heavy	84 (63%)	34 (71%)	19 (76%)	12 (52%)	11 (69%)	7 (44%)	1 (20%)	
Light	49 (37%)	14 (29%)	6 (24%)	11 (48%)	5 (31%)	9 (56%)	4 (80%)	
Cannabis history - no.(%)	20 (61%)	6 (50%)	4 (44%)	3 (75%)	5 (100%)	1 (50%)	1 (100%)	0.27
Alcohol history - no.(%)	26 (72%)	9 (69%)	5 (56%)	5 (83%)	4 (100%)	2 (67%)	1 (100%)	0.68
Commorbidities - no.(%)								
Cardiac	11 (7.6%)	2 (4.0%)	1 (3.2%)	6 (23%)	0 (0%)	1 (5.9%)	1 (20%)	
Endocrine	9 (6.2%)	3 (6.0%)	1 (3.2%)	1 (3.8%)	3 (19%)	1 (5.9%)	0 (0%)	
Pulmonary	15 (10%)	6 (12%)	4 (13%)	3 (12%)	0 (0%)	2 (12%)	0 (0%)	
RAS	110 (76%)	39 (78%)	25 (81%)	16 (62%)	13 (81%)	13 (76%)	4 (80%)	
Family history - no.(%)								0.9
Yes	9 (100%)	4 (100%)	1 (100%)	2 (100%)	1 (100%)	1 (100%)	0 (NA%)	
Histologic type - no.(%)								0.4
Adenosquamous	2 (1.4%)	0 (0%)	1 (3.2%)	1 (3.8%)	0 (0%)	0 (0%)	0 (0%)	
ADC	104 (72%)	35 (70%)	19 (61%)	19 (73%)	10 (62%)	17 (100%)	4 (80%)	
Squamous	27 (19%)	12 (24%)	9 (29%)	2 (7.7%)	4 (25%)	0 (0%)	0 (0%)	
NOS	12 (8.3%)	3 (6.0%)	2 (6.5%)	4 (15%)	2 (12%)	0 (0%)	1 (20%)	
Stage of the disease at diagnosis - no.(%)								
IB	1 (0.7%)	1 (2.0%)	0 (0%)	0 (0%)	0 (0%)	0 (0%)	0 (0%)	
IIB	4 (2.8%)	1 (2.0%)	1 (3.2%)	2 (7.7%)	0 (0%)	0 (0%)	0 (0%)	
IIIA	20 (14%)	8 (16%)	4 (13%)	1 (3.8%)	5 (33%)	1 (5.9%)	1 (20%)	
IIIB	20 (14%)	7 (14%)	2 (6.5%)	6 (23%)	1 (6.7%)	2 (12%)	2 (40%)	
IIIC	6 (4.2%)	4 (8.0%)	2 (6.5%)	0 (0%)	0 (0%)	0 (0%)	0 (0%)	
IVA	52 (36%)	21 (42%)	11 (35%)	7 (27%)	6 (40%)	6 (35%)	1 (20%)	
IVB	41 (28%)	8 (16%)	11 (35%)	10 (38%)	3 (20%)	8 (47%)	1 (20%)	
ECOG PS - no.(%)								0.50
1	125 (86%)	42 (84%)	26 (84%)	21 (81%)	14 (88%)	17 (100%)	5 (100%)	
2	20 (14%)	8 (16%)	5 (16%)	5 (19%)	2 (12%)	0 (0%)	0 (0%)	
Prior Radiotherapy - no.(%)	46 (31.7%)	18 (100%)	11 (100%)	5 (100%)	9 (100%)	3 (100%)	0 (NA%)	
Initial liver metastasis - no.(%)	18 (100%)	3 (100%)	3 (100%)	4 (100%)	2 (100%)	6 (100%)	0 (NA%)	
Initial adrenal metastasis - no.(%)	29 (100%)	7 (100%)	10 (100%)	4 (100%)	3 (100%)	4 (100%)	1 (100%)	
Initial bone metastasis - no.(%)	39 (100%)	11 (100%)	10 (100%)	8 (100%)	4 (100%)	5 (100%)	1 (100%)	
Initial brain metastasis - no.(%)	19 (13%)	5 (10%)	2 (6.5%)	6 (23%)	1 (6.2%)	4 (24%)	1 (20%)	0.23
Urgencies at diagnosis								
ICHT	1 (0.7%)	0 (0%)	0 (0%)	0 (0%)	0 (0%)	0 (0%)	1 (20%)	
RAS	104 (72%)	35 (70%)	23 (74%)	21 (81%)	12 (75%)	10 (59%)	3 (60%)	
PE	35 (24%)	14 (28%)	8 (26%)	5 (19%)	3 (19%)	4 (24%)	1 (20%)	
SVCS	5 (3.4%)	1 (2.0%)	0 (0%)	0 (0%)	1 (6.2%)	3 (18%)	0 (0%)	
Progressed Site - no.(%)								
Lung	36 (25%)	13 (26%)	7 (23%)	10 (38%)	3 (19%)	3 (18%)	0 (0%)	0.50
Liver	7 (4.8%)	3 (6.0%)	0 (0%)	1 (3.8%)	1 (6.2%)	2 (12%)	0 (0%)	0.48
Adrenal	8 (5.5%)	2 (4.0%)	1 (3.2%)	3 (12%)	0 (0%)	2 (12%)	0 (0%)	0.47
Bone	15 (10%)	6 (12%)	2 (6.5%)	2 (7.7%)	1 (6.2%)	3 (18%)	1 (20%)	0.69
Brain	8 (5.5%)	7 (14%)	0 (0%)	0 (0%)	0 (0%)	0 (0%)	1 (20%)	0.017
Events - no.(%)								
Clinic Progression	15 (10%)	5 (10%)	4 (13%)	4 (15%)	0 (0%)	2 (12%)	0 (0%)	
Control	9 (6.2%)	2 (4.0%)	4 (13%)	0 (0%)	2 (12%)	1 (5.9%)	0 (0%)	
Death	38 (26%)	10 (20%)	9 (29%)	8 (31%)	5 (31%)	3 (18%)	3 (60%)	
Partial Response	2 (1.4%)	2 (4.0%)	0 (0%)	0 (0%)	0 (0%)	0 (0%)	0 (0%)	
Lost Follow-up	25 (17%)	8 (16%)	5 (16%)	3 (12%)	6 (38%)	3 (18%)	0 (0%)	
Radiologic Progression	56 (39%)	23 (46%)	9 (29%)	11 (42%)	3 (19%)	8 (47%)	2 (40%)	
Number of cures – Median (IQR)	3.00 (2.00, 6.00)	4.00 (3.00, 6.00)	4.00 (2.00, 6.00)	3.00 (2.00, 4.00)	3.00 (2.00, 5.00)	3.00 (2.00, 5.00)	3.00 (2.50, 4.50)	0.54

*^1^* n (%); Median (IQR)

*^2^* Fisher's exact test; Kruskal-Wallis rank sum test

NSCLC, Non-Small Cell Lung Cancer; NOS, Not Otherwise Specified; ICHT, Intracranial Hypertension; PE, Pleural Effusion; SVC, Superior Vena Cava; ECOG PS, Eastern Cooperative Oncology Group Performance Status; ADC, Adenocarcinoma.

While in the SCLC patient group, 1 of 10 received docetaxel, 5 of 10 received irinotecan, 2 of 10 received etoposide, 1 of 10 received navelbine, and 1 of 10 received paclitaxel. Led by male patients (8 of 10), being heavy smokers 5 (56%), and no particular comorbidity sign (Charlson score = 0), diagnosed initially at stage IVA 4 (40%) with the presence of pleural effusion at 4 (40%) of all cases. The median age at diagnosis for the group was 58 [IQR: 52, 63]. Following neo/adjuvant or palliative first-line platinum doublet chemotherapy, the common progression site was the lung. Approximately 3 (30%) had investigator-reported bone metastases at the time of screening, 1 (10%) had liver metastasis, and 2 (20%) presented with brain metastasis. The same percentage of patients have previously undergone local brain irradiation. At the database lock (December 31, 2022), no patient from this group was continuing treatment. The median duration of prior second-line treatment and progression was 1.18 [IQR: 0.81, 2.49] months. The median duration between the last cycle of the first-line treatment and the beginning of second-line treatments was 2.7 [IQR: 0.7, 5.1] months. The median follow-up for the SCLC group was 4.4 [IQR: 1.3, 7] months (**Table 2**).

**Table 2:** SCLC patient characteristics.

Variables	Overall, N = 10*^1^*	Treatment Received					p-value*^2^*
		Docetaxel, N = 1*^1^*	Etoposide, N = 2*^1^*	Irinotecan, N = 5*^1^*	Vinorelbine, N = 1*^1^*	Paclitaxel, N = 1*^1^*	
Sex - no.(%)							>0.99
Female	2 (20%)	0 (0%)	1 (50%)	1 (20%)	0 (0%)	0 (0%)	
Male	8 (80%)	1 (100%)	1 (50%)	4 (80%)	1 (100%)	1 (100%)	
Prior First-line regimens based platinum - no.(%)							0.30
Etoposide	9 (90%)	0 (0%)	2 (100%)	5 (100%)	1 (100%)	1 (100%)	
Vinorelbine	1 (10%)	1 (100%)	0 (0%)	0 (0%)	0 (0%)	0 (0%)	
Age at diagnosis - yr Median (IQR)	54 (48, 60)	51 (51, 51)	42 (28, 63)	48 (44, 60)	59 (59, 59)	61 (61, 61)	0.3
Tobacco status - no.(%)							0.71
Never	3 (30%)	0 (0%)	1 (50%)	2 (40%)	0 (0%)	0 (0%)	
Current / Former	7 (70%)	1 (100%)	1 (50%)	3 (60%)	1 (100%)	1 (100%)	
Smoking status - no.(%)							0.71
Heavy	5 (56%)	1 (100%)	1 (50%)	1 (25%)	1 (100%)	1 (100%)	
Light	4 (44%)	0 (0%)	1 (50%)	3 (75%)	0 (0%)	0 (0%)	
Cannabis history - no.(%)	1 (25%)	0 (0%)	0 (0%)	1 (33%)	0 (0%)	0 (0%)	>0.99
Alcohol history - no.(%)	2 (40%)	0 (0%)	0 (0%)	2 (50%)	0 (0%)	0 (0%)	>0.99
Comorbidities - no.(%)							>0.99
Endocrine	2 (20%)	0 (0%)	0 (0%)	2 (40%)	0 (0%)	0 (0%)	
No	8 (80%)	1 (100%)	2 (100%)	3 (60%)	1 (100%)	1 (100%)	
Family history - no.(%)	0 (0%)	0 (0%)	0 (0%)	0 (0%)	0 (0%)	0 (0%)	
Stage of the disease at diagnosis - no.(%)							0.42
IIIA	2 (20%)	0 (0%)	1 (50%)	0 (0%)	1 (100%)	0 (0%)	
IIIB	1 (10%)	0 (0%)	0 (0%)	1 (20%)	0 (0%)	0 (0%)	
IVA	4 (40%)	1 (100%)	1 (50%)	1 (20%)	0 (0%)	1 (100%)	
IVB	3 (30%)	0 (0%)	0 (0%)	3 (60%)	0 (0%)	0 (0%)	
Urgencies at diagnosis - no.(%)							0.42
ICHT	1 (10%)	1 (100%)	0 (0%)	0 (0%)	0 (0%)	0 (0%)	
No	3 (30%)	0 (0%)	1 (50%)	1 (20%)	1 (100%)	0 (0%)	
PE	4 (40%)	0 (0%)	0 (0%)	3 (60%)	0 (0%)	1 (100%)	
SVCS	2 (20%)	0 (0%)	1 (50%)	1 (20%)	0 (0%)	0 (0%)	
ECOG PS - no.(%)							>0.99
1	9 (90%)	1 (100%)	2 (100%)	4 (80%)	1 (100%)	1 (100%)	
2	1 (10%)	0 (0%)	0 (0%)	1 (20%)	0 (0%)	0 (0%)	
Prior Radiotherapy - no.(%)	5 (50%)	0 (0%)	1 (50%)	3 (66%)	0 (0%)	1 (100%)	
Initial liver metastasis - no.(%)	1 (100%)	0 (0%)	0 (0%)	1 (20%)	0 (0%)	0 (0%)	
Initial adrenal metastasis - no.(%)	0 (0%)	0 (0%)	0 (0%)	0 (0%)	0 (0%)	0 (0%)	
Initial bone metastasis - no.(%)	3 (100%)	0 (0%)	0 (0%)	3 (60%)	0 (0%)	0 (0%)	
Initial brain metastasis - no.(%)	2 (20%)	1 (100%)	0 (0%)	1 (20%)	0 (0%)	0 (0%)	0.56
Progressed Site - no.(%)							0.089
Lung	2 (20%)	0 (0%)	0 (0%)	0 (0%)	1 (100%)	1 (100%)	
Liver	0 (0%)	0 (0%)	0 (0%)	0 (0%)	0 (0%)	0 (0%)	
Adrenal	0 (0%)	0 (0%)	0 (0%)	0 (0%)	0 (0%)	0 (0%)	
Bone	0 (0%)	0 (0%)	0 (0%)	0 (0%)	0 (0%)	0 (0%)	
Brain	0 (0%)	0 (0%)	0 (0%)	0 (0%)	0 (0%)	0 (0%)	
Events - no.(%)							0.60
Control	2 (20%)	0 (0%)	1 (50%)	1 (20%)	0 (0%)	0 (0%)	
Death	5 (50%)	1 (100%)	1 (50%)	3 (60%)	0 (0%)	0 (0%)	
Lost Follow-up	1 (10%)	0 (0%)	0 (0%)	1 (20%)	0 (0%)	0 (0%)	
Radiologic Progression	2 (20%)	0 (0%)	0 (0%)	0 (0%)	1 (100%)	1 (100%)	

*^1^* n (%); Median (IQR)

*^2^* Fisher's exact test; Kruskal-Wallis rank sum test

SCC, Small Cell Carcinoma; LNEC, Lung Neuroendocrine Carcinoma; ICHT, Intracranial Hypertension; PE, Pleural Effusion ; SVC, Superior Ve0 Cava; ECOG PS:

The smoking status was defined based on the optimal cut-off value obtained by the ROC results from the cohort, given that patients with less than or equal to 20 PA were light smokers, while patients with more than 20 PA were defined as heavy smokers.

### Efficacy

#### Overall survival

**Figure 1:**
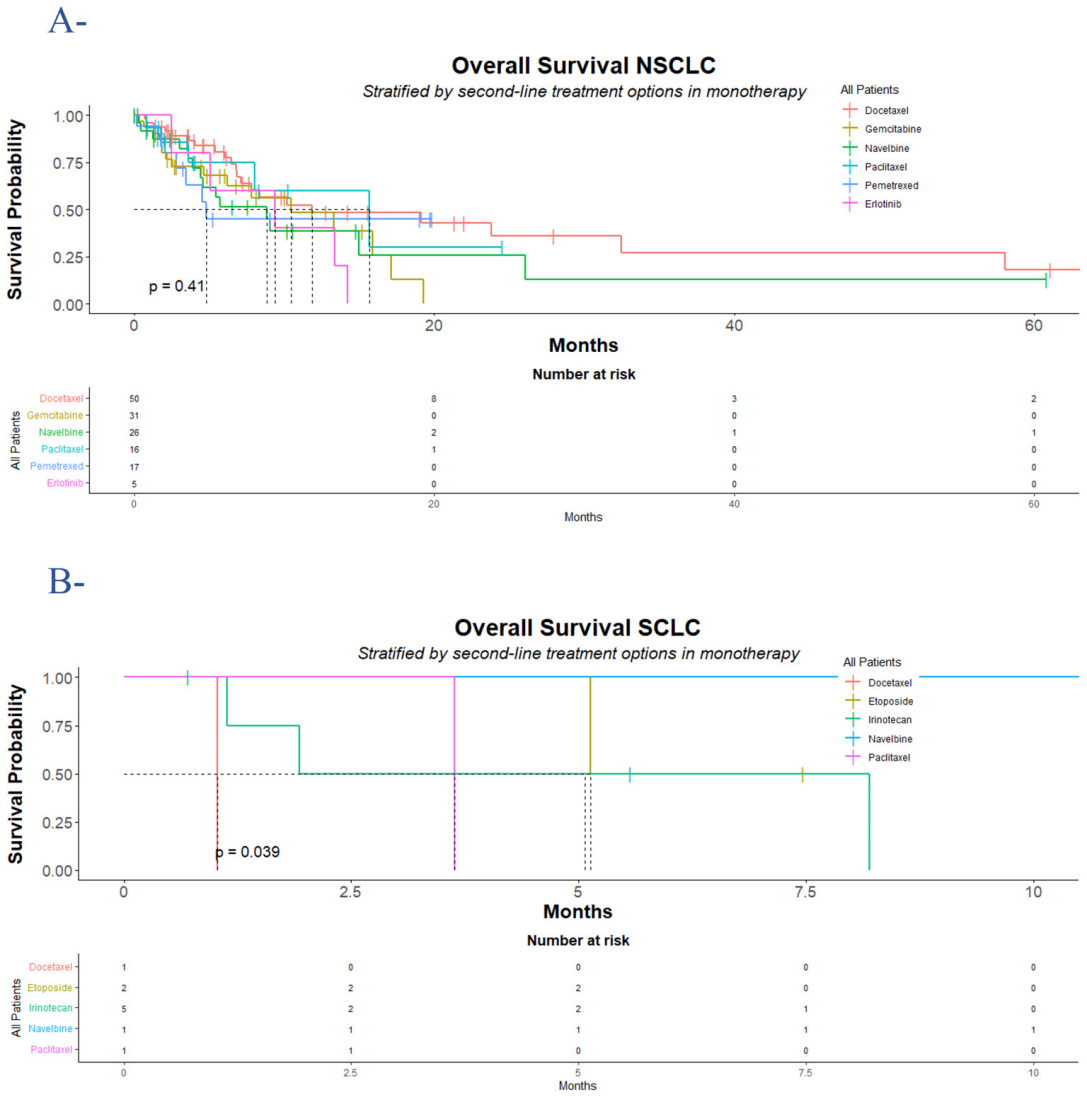
Overall survival for NSCLC and SCLC population who progressed following platinum-doublet chemotherapy. According to treatment group, Kaplan-Meier estimates of overall survival for the NSCLC population (A) and the SCLC population (B) are displayed. In the NSCLC population, 68 (47%) cases of death were observed, divided into 21 (42%) of 50 in the docetaxel group, 16 (52%) of 31 in the gemcitabine group, 14 (54%) of 26 in the vinorelbine group, 5 (31%) of 16 in the paclitaxel group, 7 (41%) of 17 in the pemetrexed group, and 5 (100%) of 5 in the erlotinib group. In the SCLC population, 6 (60%) of 10 cases of death were observed, divided into 1 (100%) of 1 in the docetaxel group, 1 (50%) of 2 in the etoposide group, 3 (60%) of 5 in the irinotecan group, and 1 (100%) of 1 in the paclitaxel group. The difference between the treatment groups was determined with a log-rank test. NSCLC denotes non-small cell lung cancer; and SCLC denotes small cell lung cancer. Censored data were presented with tick marks.

For NSCLC patients, at the time of analysis, 68 (47%) cases of death were observed; 71 cases occurred after the second clinic or radiologic progression. The median OS was 10 (95% CI: 7.8, 16) months (hazard ratio for death: 1.49; 95% CI: 0.63, 3.54; p = 0.4). The median OS was slightly higher in the group of patients who received paclitaxel 16 (95% CI: 8, Not Estimable [NE]) months, compared to docetaxel group 12 (95% CI: 7.1, NE) months, gemcitabine group 10 (95% CI: 6.2, NE) months, erlotinib group 9.4 (95% CI: 5.1, NE) months, vinorelbine group 8.9 (95% CI: 4.4, NE) months, and pemetrexed group (95% CI: 3.4, NE) months. While the estimated 18-month OS rate was a little higher with docetaxel 48% (95% CI, 33-70) and pemetrexed 45% (95% CI: 24-84) compared to paclitaxel 30% (95% CI: 6.7-100), vinorelbine 26% (95% CI: 9.5-69), and gemcitabine 13% (95% CI: 2.3-72), the 18-month OS rate was not reached [NR] in the erlotinib group NR (95% CI: NE-NE). The death events were seen in 5 (100%) in the erlotinib group, 14 (54%) in the vinorelbine group, 16 (52%) in the gemcitabine group, 21 (42%) in the docetaxel group, 7 (41%) in the pemetrexed group, and 5 (31%) in the paclitaxel group (**Figure 1A**, **Table 1**).

In SCLC patients, 6 (60%) death events were recorded; two of them occurred after radiologic progression. The median OS for the entire group was 5.1 (95% CI: 1.9, NE) months (hazard ratio for death: NE; p = 0.03). Overall, etoposide showed acceptable activity over irinotecan, paclitaxel, and docetaxel, respectively, with a median time to death of 6.3 (95% CI: 5.1, NE) months, 5.1 (95% CI: 1.1, NE) months, 3.6 (95% CI: NE, NE) months, and 1.0 (95% CI: NE, NE) months, respectively, while in the vinorelbine group the median OS was not reached. The 12-month and 18-month OS rates were never reached in the entire group of treatments. In the group of patients who received irinotecan, 3 of 5 patients died, 1 over 2 died in the etoposide group, while patients who received docetaxel and paclitaxel died during treatment (**Figure 1B**, **Table 2**).

#### Progression-free survival

**Figure 2:**
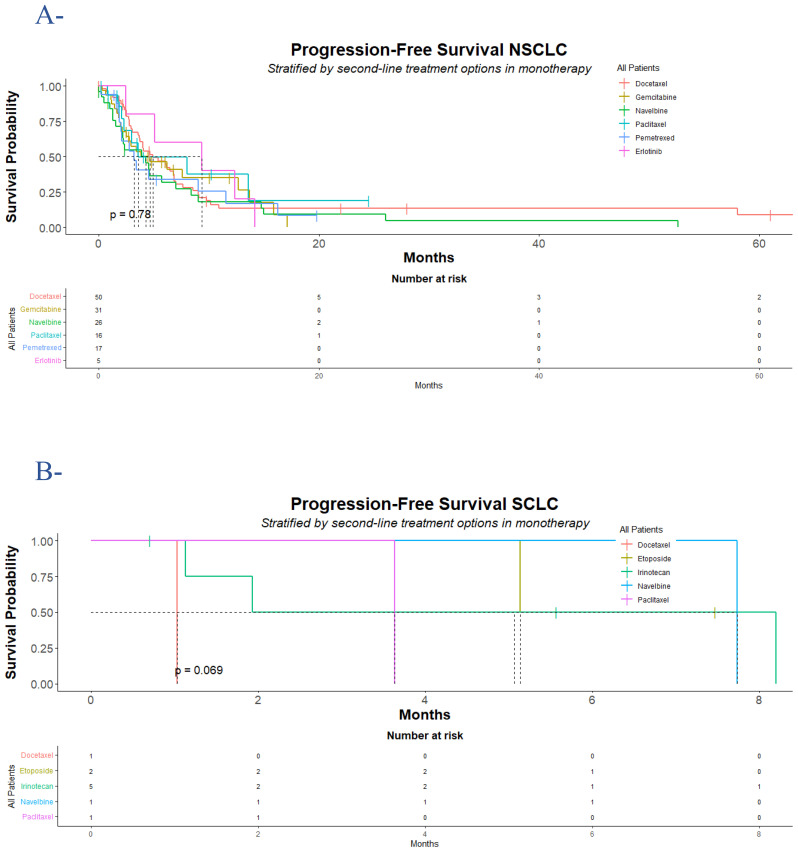
Progression-free survival for NSCLC and SCLC populations who progressed following platinum-doublet chemotherapy. The data displayed are as of December 31, 2022, which was the cutoff date for the analysis. In the NSCLC population (A), a total of 110 (76%) of 145 had documented disease progression according to Response Evaluation Criteria in Solid Tumours (RECIST), version 1.1, divided into 39 (78%) in the docetaxel group, 22 (71%) in the gemcitabine group, 23 (88%) in the vinorelbine group, 8 (50%) in the paclitaxel group, 13 (76%) in the pemetrexed group, and 5 (100%) in the erlotinib group. In the SCLC population (B), a total of 7 (70%) of 10 had documented disease progression according to Response Evaluation Criteria in Solid Tumours (RECIST), version 1.1, divided into 1 (100%) in the docetaxel group, 1 (50%) in the etoposide group, 3 (60%) in the irinotecan group, 1 (100%) in the vinorelbine group, and 1 (100%) in the paclitaxel group. The difference between the treatment groups was determined with a log-rank test. NSCLC denotes non-small cell lung cancer; and SCLC denotes small cell lung cancer. Censored data were presented with tick marks.

Regarding the NSCLC group, at the data cutoff, a total of 109 events of interest (progression or death) were recorded, divided into 15 (10%) clinical progression, 56 (39%) radiologic progression as assessed by RECIST 1.1 criteria, and 38 (26%) death cases recorded during second-line treatments. The median PFS for the entire group was 4.5 (95% CI: 3.6, 6.2) months (hazard ratio for progression or death for docetaxel vs. gemcitabine vs. vinorelbine vs. paclitaxel vs. pemetrexed vs. erlotinib: 1.1; 95% CI: 0.65, 1.86; p = 0.78). The median time to progression was higher in the erlotinib group 9.4 (95% CI: 5.1, NE) months compared to 4.9 (95% CI: 3.7, 7) months in the docetaxel group, 4.6 (95% CI: 2.5, NE) months in the gemcitabine group, 4.3 (95% CI: 2.2, 8.4) months in the vinorelbine group, 3.6 (95% CI: 2.3, NE) months in the paclitaxel group, and 3.2 (95% CI: 2, NE) months in the pemetrexed group. The 12-month PFS rate was higher in the erlotinib group 40% (95% CI: 14-100), followed by 37% (95% CI: 17-84) in the paclitaxel group, 35% (95% CI: 20-61) in the gemcitabine group, 18% (95% CI: 7.5-44) in the vinorelbine group, 17% (95% CI: 5-57) in the pemetrexed group, and 13% (95% CI: 6.1-29) in the docetaxel group. In the docetaxel group of patients, lung was the most progressive site 13 (26%) followed by brain 7 (14%), bone 6 (12%), liver 3 (6%) and adrenal 2 (4%) respectively. In the gemcitabine group of the population, the incidence of lung progression was higher in 7 (23%), followed by bone and adrenal in 2 (6.5%) and 1 (3.2%), respectively. In the vinorelbine group, lung was the primary progression site in 10 (38%) of patients, followed by adrenal in 3 (12%), bone in 2 (7.7%), and liver in all cases. Again, lung was predominantly the non-responder site in the paclitaxel and pemetrexed groups, followed by bone in 19% and 18% of all cases, respectively (**Figure 2A**, **Table 1**).

Concerning the SCLC patients, a total of two radiological progressions were noticed, and five cases of death were recorded during second-line treatments. The median time to progression for the entire group was 5.1 (95% CI: 1.9, NE) months (hazard ratio for progression or death for docetaxel vs. etoposide vs. vinorelbine vs. paclitaxel vs. irinotecan: NE; p = 0.06). The median PFS was higher in the vinorelbine group with 7.7 (95% CI: NE, NE) months and etoposide 6.3 (95% CI: 5.1, NE) months compared to irinotecan 5.1 (95% CI: 1.1, NE) months, paclitaxel 3.6 (95% CI: NE, NE) months, and docetaxel 1 (95% CI: NE, NE) months. At 12 months, the PFS rate was not reached in all groups of patients. Lung was the most noticed progressed site, and in 2 (20%) of all cases, no other progressed site was recorded (**Figure 2B**, **Table 2**).

Of note, due to treatment momentum inaccessibility, 8 (5.2%) LC patients switched to second-line therapy, 4 (2.6%) of them in the docetaxel arm, 2 (1.3%) in the pemetrexed group, and two in the gemcitabine and navelbine groups. Moreover, no patient has received radiotherapy prior to, during, or after the second line.

#### Clinical benefit

Overall, at the follow-up, the overall response rate (ORR) was assessed in the platinum-based chemotherapy-relapsed patients with zero complete response (CR), two partial responses (PRs), 9 (6.2%) cases of disease control (DC) in the NSCLC population group, and 2 (20%) DC cases in the SCLC group of the population; therefore, the objective response rate was never achieved (**Tables 1**, **2**).

The two PRs were observed for the NSCLC population under docetaxel after six cycles. while most control cases were observed who had received gemcitabine 4 (13%), docetaxel 2 (4%), 2 (12%) in the paclitaxel group, and 1 (5.9%) in the pemetrexed group, in a median time-to-control of 4.5 months in the gemcitabine group, 4.5 months in the docetaxel group, 3.7 months in the paclitaxel group, and 4 months in the pemetrexed group. The two DC cases in the SCLC population were observed in the irinotecan and etoposide groups for a duration time to response of 5.2 months.

### Safety

Of note, all subgroups of patients who received at least one dosage of various therapies underwent hematologic safety analysis. The frequency of hematologic adverse events considered to be related to treatments of any grade was reported by fewer patients.

In the NSCLC population, the median duration of treatments in the docetaxel group was 4 [IQR: 2, 8] months, 3 [IQR: 2, 6] months in the gemcitabine group, 2 [IQR: 1, 7] months in the vinorelbine group, 3 [IQR: 2, 5] months in the pemetrexed group, and 9 [IQR: 5, 12] months in the erlotinib group. Grades 3–4, anemia, neutropenia, and thrombocytopenia were less frequent (26 (18%), 16 (11%), and 13 (9%), respectively, mostly reported in the docetaxel and vinorelbine groups (**Supplementary Table S2**). While in the SCLC population, the median duration of treatments in the docetaxel group was 1.03 [IQR: 1.03, 1.03] months, 6.3 [IQR: 5.7, 6.9] months in the etoposide group, 1.9 [IQR: 1.1, 5.6] months in the irinotecan group, 7.7 months in the vinorelbine group, and 3.6 in the paclitaxel group. Grade 3–4 anemia, neutropenia, and thrombocytopenia were reported in 4 (40%), 3 (30%), and 1 (10%) of all cases, mainly occurring in the irinotecan patient group (**Supplementary Table S3**). Due to adverse events related to second-line treatments, discontinuation occurred in all patients who experienced grade 4 anemia, neutropenia, and thrombocytopenia.

Two deaths in the docetaxel group were assessed by clinicians at that time as being related to heart attacks and acute kidney failure, all in the NSCLC population. Other cases of death were regarded as unknown since patients were only traced using their phone numbers in the two populations.

## Discussion

Patients with NSCLC receive second-line ICI as a part of the standard of care after failure of first-line platinum-based chemotherapy (23, 24), regardless of whether or not maintenance therapy is administered, while in patients with SCLC, the standard of care still includes chemotherapy with platin and etoposide (25–27) or lurbinectedin (28) after failure of first-line platinum-chemotherapy as an option in second-line settings (29). Additionally, salvage treatment with docetaxel following 1L chemotherapy only provides a 14% overall response rate (ORR) (30), underlining the need for additional treatments that can extend disease control.

Through this study, we aimed to compare accessible treatments as the second line for all LC subcategories based on real-life data. The medical oncology database included nearly 1371 consecutive LC patients with an identical sex ratio and a different median age at diagnosis from the general population of Moroccan LC patients presented by the International Agency for Research on Cancer in 2020 (31).

The results relied on 155 patients presenting different histologic types in various stages (IB-IVB) diagnosed between January 2013 and December 2022. Their traits were remarkably compatible with demographic data from throughout the world (32). Dominated by current smoker males with a median age of 58 years, often diagnosed with NSCLC at late stage IVA, the majority received chemotherapy, and only five were treated with erlotinib and were not selected according to EGFR status.

To our knowledge, this is the first head-to-head study of its kind in the region and continent that evaluates real-world outcomes after a long-term follow-up, providing insights into the prognosis of LC patients who have previously been treated with up-front doublet chemotherapy-based platinum in regular practice is mainly based on several parameters, regardless of the initial stage and ECOG PS. With challenging second-line chemotherapy, we provided evidence of an acceptable period of PFS and OS times. This entails that following immunotherapy advancement, there may be differences between African patients and those reported in real-world studies and those participating in clinical trials who received ICIs, TKIs, or antiangiogenics in monotherapy or combination with chemotherapy.

At the data cut-off, the median PFS was nearly equivalent between all treatment groups (hazard ratio for progression or death for docetaxel vs. gemcitabine vs. vinorelbine vs. paclitaxel vs. pemetrexed vs. erlotinib: 1.1; 95% CI: 0.65, 1.86; p = 0.78). Similar to PFS, we noticed no differences between treatment groups in terms of proving OS (hazard ratio for death for docetaxel vs. gemcitabine vs. vinorelbine vs. paclitaxel vs. pemetrexed vs. erlotinib: 1.49; 95% CI: 0.63, 3.54; p = 0.4). While prior chemotherapy may impact the efficacy of later agents, the obtained results suggest that, whatever previous regimens were received, there was no statistical significance between treatment groups. Because of the small sample sizes in both the NSCLC and SCLC populations, stratification by sex (male or female) and smoking status (light, heavy) was not carried out for each treatment group.

The prognosis for patients with relapsed SCLC is extremely poor. One of the key variables affecting subsequent clinical outcomes in the relapsed scenario is the depth and, more importantly, the durability of the response to platinum-based therapy. Having increasing disease as the response is regarded as a refractory disease, and a 90-day cut-off value of the first response to platinum-based chemotherapy distinguishes between sensitive (more than 90 days) and resistant disease (less than 90 days) (29). A pooled review of twenty-one research papers in recurrent second-line SCLC found that sensitive individuals had significantly better median OS (7.7 months) than refractory patients (5.4 months) (33). The median refractory time related to our SCLC population was 4.4 (range: 1.3 - 7) months, which implies the existence of both categories considered to have a resistant and sensitive disease, and due to the small sample size (n = 10), we chose not to segregate according to relapse time. While vinorelbine was associated with better PFS (7.7 months), etoposide in monotherapy was associated with slightly less median PFS and OS (6.3 months) among our patients, contrary to those who received irinotecan (5.1 months), which is approximately the same obtained with carboplatin plus etoposide rechallenge (4.7 months), lurbinectedin (4.6 months) for sensitive patients, and less than what have the basket trial (2.6 months with lurbinectedin) for resistant patients, all pre-treated with platin-doublet chemotherapy (26, 28).

Since ICIs are the gold standard of care in both squamous and non-squamous histology in refractory patients pre-treated with doublet chemotherapy-based platinum in the first line proving a long-term benefit with less hematologic toxicities, it ought to be noted that in our case when access to the standard is such an issue, docetaxel proved to be associated with acceptable OS compared to the results obtained with checkMate 017 (24), checkMate 057 (23), OAK (34) studies, regardless PDL-1 status, and the reported outcomes with CONTACT-01 (35) obtained with docetaxel after failure of first-line ICIs plus chemotherapy. The addition of pembrolizumab to docetaxel, as assessed by the PROLUNG study, for patients without targetable drivers showed superiority over docetaxel alone, offering a 9.5-month vs. 3.9-month PFS with an ORR of 42.5% vs. 15.8%, respectively (36). The efficacy and safety results with the combination of ICI and docetaxel in previously treated patients with platin-based chemotherapy were seen in patients treated with the combination of sintilimab plus docetaxel with a PFS of 5.8 months (37, 38).

Although patients with a variety of solid tumors benefit clinically from ICI therapy, an EGFR TKI sold in Morocco under Tarceva has proved its utility for unknown EGFR status patients (39). We should note that erlotinib is still not publicly available in Morocco, and only those who can cover the fees can benefit. In a phase 3 TITAN study conducted by Tudor Ciuleanu and his colleagues (40), comparing erlotinib and investigators choice between docetaxel and pemetrexed, they found no significant difference between erlotinib and chemotherapy arm in proving OS for naïve chemotherapy patients or pre-treated with platin-based chemotherapy regardless of EGFR status. In our case, erlotinib in the second line was associated with better PFS in NSCLC population, with a median PFS of 9.4 months, but docetaxel and paclitaxel were associated with better OS for the same patients’ subcategories successively (12 months and 16 months).

Comparing the results reported on challenging chemotherapy through this study with the outcome reported with an antiangiogenic plus chemotherapy remains important but not necessary since it is recognized as an option of treatment for pretreated patients. The combination with ramucirumab (30) or nintedanib (41) did not improve a long PFS compared to what immunotherapy in monotherapy offered and the superiority presented. In contrast, the combination of ICI and antiangiogenic inhibitors, such as atezolizumab and bevacizumab, has demonstrated encouraging anticancer efficacy and an acceptable safety profile in patients with advanced NSCLC who relapsed from first-line platinum-containing treatment (42).

Yet, no prospective phase III study in NSCLC patients without actionable genomic alterations has explicitly addressed the chemotherapy rechallenge either with or without platinum-based regimens. In addition, no head-to-head prospective study has been conducted to compare the outcome of gemcitabine with other chemotherapy agents. In a retrospective study comparing the outcome of docetaxel and gemcitabine in Turkish patients who relapsed in first-line under platin plus chemotherapy, an improvement in PFS was found in the gemcitabine arm, while a similarity was found with OS, and the response over one year was less favorable with gemcitabine 8% than docetaxel 18% (43). Nevertheless, the results obtained with OS are doubtable since the methodological approach adopted with the calculation remains unclear. A significant prolonged PFS was seen with gemcitabine in monotherapy compared with what vinorelbine and pemetrexed can offer in the same cohort. In addition, the median OS was observed with paclitaxel (16 months), which was higher compared to what nivolumab offered in non-squamous patients in checkmate 057 and real-world data (44–47).

Generally, we assessed PFS as shorter with SCLC histology as well as OS than with NSCLC in this study. Mostly because of the ineffectiveness of the available drugs, PFS and OS could be assessed while ORR cannot. Overall, treatment with docetaxel resulted in two partial responses observed in NSCLC population, while disease control was mostly associated with gemcitabine, paclitaxel and docetaxel, respectively. Due to 10 years of follow-up, we could unfortunately not judge the superiority or inferiority of docetaxel over another chemo-agent such as gemcitabine or navelbine in NSCLC patients since we assessed a significant one-year survival efficacy and hematologic safety profiles in the tree groups.

The incidence of hematologic-related adverse events observed with cytotoxic chemotherapy or taxan agents was substantially low; they frequently appeared in grades 1–2 anemia, respectively, and resulted in docetaxel, with no recorded severity. Overall, a few severe grade 4 chemotherapy-related adverse events were seen that resulted in treatment discontinuation. No new safety profile was identified. Indeed, the hematologic safety of the assessed chemotherapy was correlated with the drug profiles. However, the recorded deaths could not be attributed to treatments since most were not assessed by clinicians at the time of occurrence. Generally, the incidence of death was approximately equal between the groups of patients. The survival benefit that was generally observed with docetaxel, gemcitabine for NSCLC, and with vinorelbine and etoposide for SCLC in pre-treated patients. Currently, with the introduction of a national health insurance agency earlier this year, whose objective is to guarantee medical coverage and consolidate the rights acquired by Moroccan citizens benefiting from health insurance, our patients will now have access to the majority of drugs as recommended by the guidelines; thus, further studies will now be directed based on novel recommendations to assess the efficacy and tolerability of these drugs with a proportion of African patients.

### Study limitations

This study must be viewed in light of its limitations. Due to the fact that this study was retrospective and lacked a control group, historical comparisons within the literature or real-world evidence are needed to interpret the data. The study’s small sample size results in less precise results than preferred, especially with etoposide in SCLC and erlotinib in NSCLC, which might affect interpretation and extrapolation. Owing to the small sample size, bias could have been introduced into the subgroup analyses across the different treatment groups, particularly with reference to the erlotinib. It was therefore decided not to include the subgroup analysis. Furthermore, the described findings applied only to resistant patients who relapsed after platinum-based chemotherapy. Additionally, the follow-up period examined in this study did not adequately account for all aspects of long-term safety, including quality of life.

When extracting information manually, the other treatment-related adverse events were formerly taken into account; however, we decided not to analyze this characteristic anymore because of the high percentage of missing values, which exceeded 97%. Due to the significant percentage of missing values—wherein the patient’s medical record has almost no information in this regard—further early, current, or late adverse events were not assessed.

## Conclusion

In conclusion, this study was the first retrospective investigation of chemotherapy in monotherapy rechallenges in NSCLC, and SCLC populations with unknown oncogenic drivers whose disease has progressed following platinum doublet chemotherapy. Despite the exclusion of effective biomarkers from stratification, the results suggest that more variables should be taken into consideration when studying the population. It could be considered a basic treatment in addition to ICI or TKI for selected patients. The data to guide treatment selection for these patients is limited and based on retrospective analyses. The results of this study evaluating old single agents are greatly needed to find alternative strategies that can improve survival outcomes for these patients in low-income countries.

## Data Availability

The data analyzed in this study is subject to the following licenses/restrictions: The datasets used and/or analyzed during the current study are available from the corresponding author on reasonable request. Requests to access these datasets should be directed to hassanabdelilah.tafenzi@gmail.com.
