# Supplementary material for: Second-line chemotherapy rechallenge in lung cancer patients: a Moroccan real-world study
Source: Front Oncol. 2025 May 22;15:1489327. doi: 10.3389/fonc.2025.1489327 (PMC12142062; doi:10.3389/fonc.2025.1489327)
Supplement: Supplementary file 1 [file SupplementaryFile1.docx]

Supplementary Appendix

The authors have produced this appendix to offer readers with further details about their findings.

Supplementary Methods

**Table S1. Dosing table**

| **Regimen** | **Dose** | **Frequency** | **Route** | **Duration** |
| --- | --- | --- | --- | --- |
| Vinorelbine | 30 mg/m² | Days 1 and 8 | Intravenous | every 3 weeks |
| Paclitaxel | 175 mg/m² | Day 1 | Intravenous | every 3 weeks |
| Docetaxel | 75 mg/m² | Day 1 | Intravenous | every 3 weeks |
| Etoposide | 100 mg/m² | Days 1, 2 and 3 | Intravenous | every 3 weeks |
| Pemetrexed | 500 mg/m² | Day 1 | Intravenous | every 3 weeks |
| Gemcitabine | 1000 mg/m² | Days 1 and 8 | Intravenous | every 3 weeks |
| *Drugs used alone in monotherapy* | | | | |

Supplementary Results

**Figure 1: Screening, Analysis, and Follow-Up**

The data displayed are as of December 31^st^, 2022, which was the cutoff date for the analysis. In the NSCLC group, 56 patients had documented disease progression according to Response Evaluation Criteria in Solid Tumours (RECIST), version 1.1; 15 had clinical progression; 25 had lost follow-up; and 38 died. In the SCLC group, 02 patients had documented disease progression according to Response Evaluation Criteria in Solid Tumours (RECIST), version 1.1; 00 had clinical progression; 01 had lost follow-up; and 05 died.


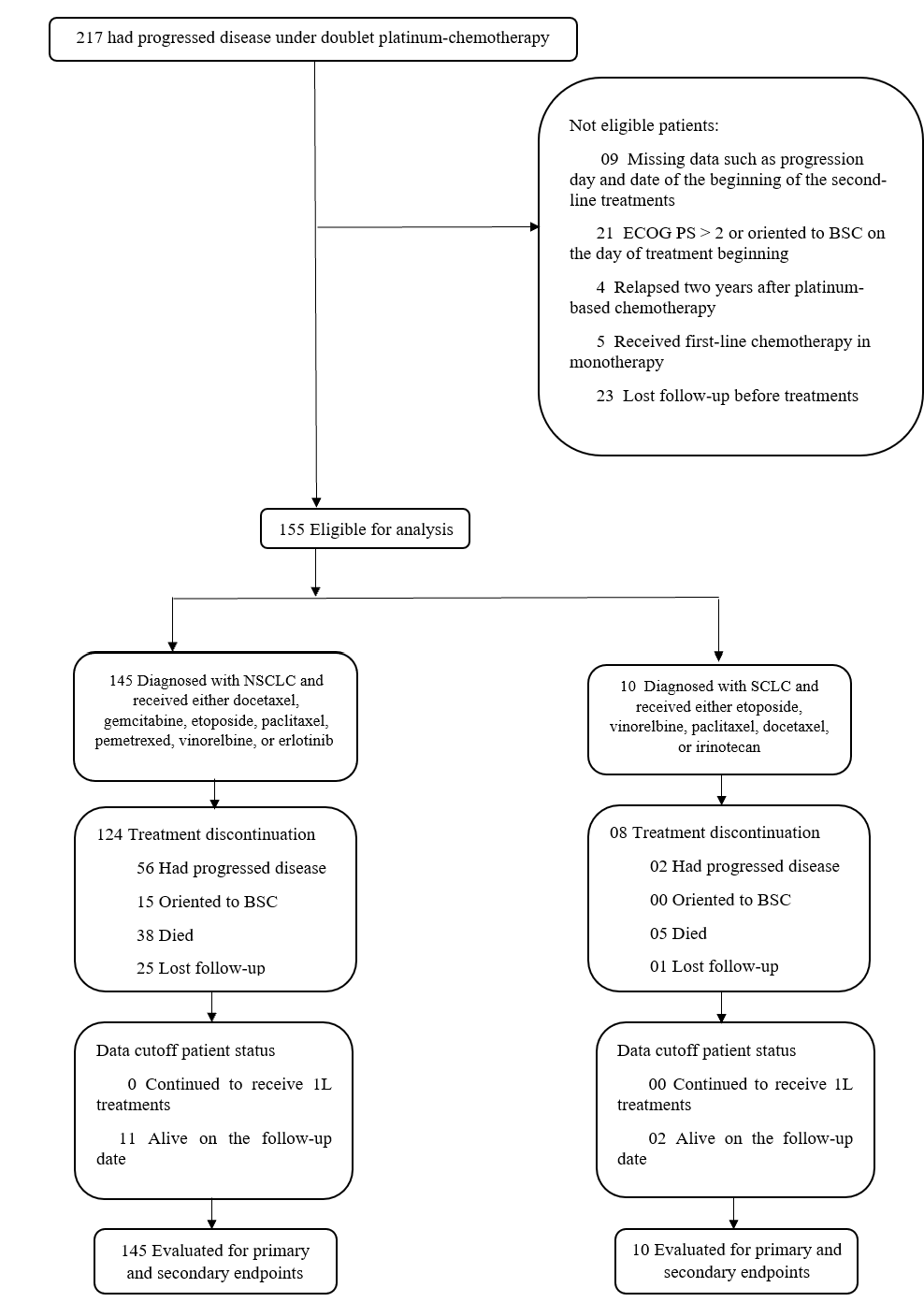


| **Table S2. Treatments Related Hematologic Adverse Events (NSCLC)** | | | | | | | | |
| --- | --- | --- | --- | --- | --- | --- | --- | --- |
| **Variables** | **Overall**, N = 145*^1^* | **Treatment Received** | | | | | | **p-value***^2^* |
|  |  | **Docetaxel**, N = 50*^1^* | **Gemcitabine**, N = 31*^1^* | **Vinorelbine**, N = 26*^1^* | **Paclitaxel**, N = 16*^1^* | **Pemetrexed**, N = 17*^1^* | **Erlotinib**, N = 5*^1^* |  |
| **Anemia** |  |  |  |  |  |  |  |  |
| G0 | 13 (15%) | 4 (12%) | 5 (25%) | 3 (21%) | 1 (11%) | 0 (0%) | 0 (0%) |  |
| G1 | 25 (28%) | 10 (31%) | 6 (30%) | 1 (7.1%) | 3 (33%) | 4 (36%) | 1 (50%) |  |
| G2 | 24 (27%) | 9 (28%) | 4 (20%) | 5 (36%) | 2 (22%) | 4 (36%) | 0 (0%) |  |
| G3 | 22 (25%) | 9 (28%) | 4 (20%) | 5 (36%) | 1 (11%) | 2 (18%) | 1 (50%) |  |
| G4 | 4 (4.5%) | 0 (0%) | 1 (5.0%) | 0 (0%) | 2 (22%) | 1 (9.1%) | 0 (0%) |  |
| **Neutropenia** |  |  |  |  |  |  |  |  |
| G0 | 21 (31%) | 4 (17%) | 9 (47%) | 5 (36%) | 2 (33%) | 1 (20%) | 0 (0%) |  |
| G1 | 22 (32%) | 7 (30%) | 3 (16%) | 6 (43%) | 3 (50%) | 3 (60%) | 0 (0%) |  |
| G2 | 9 (13%) | 4 (17%) | 5 (26%) | 0 (0%) | 0 (0%) | 0 (0%) | 0 (0%) |  |
| G3 | 10 (15%) | 6 (26%) | 1 (5.3%) | 1 (7.1%) | 0 (0%) | 1 (20%) | 1 (100%) |  |
| G4 | 6 (8.8%) | 2 (8.7%) | 1 (5.3%) | 2 (14%) | 1 (17%) | 0 (0%) | 0 (0%) |  |
| **Thrombocytopenia** |  |  |  |  |  |  |  | 0.73 |
| G0 | 30 (55%) | 10 (62%) | 11 (65%) | 5 (38%) | 3 (75%) | 1 (20%) | 0 (0%) |  |
| G1 | 8 (15%) | 3 (19%) | 2 (12%) | 2 (15%) | 0 (0%) | 1 (20%) | 0 (0%) |  |
| G2 | 4 (7.3%) | 1 (6.2%) | 1 (5.9%) | 1 (7.7%) | 0 (0%) | 1 (20%) | 0 (0%) |  |
| G3 | 8 (15%) | 1 (6.2%) | 1 (5.9%) | 3 (23%) | 1 (25%) | 2 (40%) | 0 (0%) |  |
| G4 | 5 (9.1%) | 1 (6.2%) | 2 (12%) | 2 (15%) | 0 (0%) | 0 (0%) | 0 (0%) |  |
| *^1^* n (%); Median (IQR) | | | | | | | |  |
| *^2^* Fisher's exact test; Kruskal-Wallis rank sum test | | | | | | | |  |

| **Table S2. Treatments Related Hematologic Adverse Events (SCLC)** | | | | | | | | |
| --- | --- | --- | --- | --- | --- | --- | --- | --- |
| **Variable** | **Overall**, N = 10*^1^* | **Treatment Received** | | | | | **p-value***^2^* |  |
|  |  | **Docetaxel**, N = 1*^1^* | **Etoposide**, N = 2*^1^* | **Irinotecan**, N = 5*^1^* | **Vinorelbine**, N = 1*^1^* | **Paclitaxel**, N = 1*^1^* |  |  |
| **Anemia** |  |  |  |  |  |  | 0.94 |  |
| G0 | 1 (11%) | 0 (0%) | 1 (50%) | 0 (0%) | 0 (0%) | 0 (0%) |  |  |
| G1 | 4 (44%) | 1 (100%) | 1 (50%) | 1 (25%) | 0 (0%) | 1 (100%) |  |  |
| G3 | 3 (33%) | 0 (0%) | 0 (0%) | 2 (50%) | 1 (100%) | 0 (0%) |  |  |
| G4 | 1 (11%) | 0 (0%) | 0 (0%) | 1 (25%) | 0 (0%) | 0 (0%) |  |  |
| **Neutropenia** |  |  |  |  |  |  | >0.99 |  |
| G0 | 1 (17%) | 0 (0%) | 0 (0%) | 0 (0%) | 1 (100%) | 0 (0%) |  |  |
| G1 | 1 (17%) | 0 (0%) | 0 (0%) | 1 (33%) | 0 (0%) | 0 (0%) |  |  |
| G2 | 1 (17%) | 0 (0%) | 0 (0%) | 0 (0%) | 0 (0%) | 1 (100%) |  |  |
| G3 | 1 (17%) | 0 (0%) | 0 (0%) | 1 (33%) | 0 (0%) | 0 (0%) |  |  |
| G4 | 2 (33%) | 0 (0%) | 1 (100%) | 1 (33%) | 0 (0%) | 0 (0%) |  |  |
| **Thrombocytopenia** |  |  |  |  |  |  | >0.99 |  |
| G0 | 4 (80%) | 0 (0%) | 1 (100%) | 2 (67%) | 1 (100%) | 0 (0%) |  |  |
| G4 | 1 (20%) | 0 (0%) | 0 (0%) | 1 (33%) | 0 (0%) | 0 (0%) |  |  |
| *^1^* n (%); Median (IQR) | | | | | | | |  |
| *^2^* Fisher's exact test; Kruskal-Wallis rank sum test | | | | | | | |  |
